# The role of cysteine-rich protein in enhancing mandarivirus infectivity and pathogenicity

**DOI:** 10.1128/jvi.02237-24

**Published:** 2025-05-19

**Authors:** Jiaxing Wu, Xiaofei Liang, Die Li, Xuedong Liu, Zongtao Sun, Changyong Zhou, Xuefeng Wang, Mengji Cao

**Affiliations:** 1 Integrative Science Center of Germplasm Creation in Western China (Chongqing) Science City, Citrus Research Institute, Southwest University 117459, Chongqing, China; 2 National Citrus Engineering Research Center, Chongqing, China; 3 Guangdong Provincial Key Laboratory for Plant Epigenetics, Longhua Bioindustry and Innovation Research Institute, College of Life Sciences and Oceanography, Shenzhen University 616964 , Shenzhen, China; 4 State Key Laboratory for Managing Biotic and Chemical Threats to the Quality and Safety of Agro-products, Key Laboratory of Biotechnology in Plant Protection of MARA and Zhejiang Province, Ningbo University 47862, , Ningbo, China; Iowa State University, Ames, Iowa, USA

**Keywords:** citrus yellow vein clearing virus, citrus yellow mottle-associated virus, pathogenicity determinant, RNA silencing suppressors, vacuum infiltration

## Abstract

Mandariviruses pose a significant threat to the citrus industry due to their diverse transmission modes and high pathogenicity. However, the pathogenicity mechanisms of mandariviruses remain largely unknown, especially as there is a complete lack of understanding regarding the function of the cysteine-rich proteins (CRPs) encoded by mandariviruses during infection. In this study, ectopic expression of two mandarivirus CRPs from citrus yellow mottle-associated virus (CiYMaV) and citrus yellow vein clearing virus (CYVCV) using a potato virus X vector resulted in severe symptoms and increased viral accumulation in *Nicotiana benthamiana*. CiYMaV CRP induced cell death in *N. benthamiana* leaves, with the zinc finger motif identified as the critical region responsible for this induction. CYVCV CRP functioned as a suppressor to inhibit local gene silencing induced by single-stranded *GFP*, but not double-stranded *GFP*. Furthermore, mutational analysis of the infectious clones of CiYMaV and CYVCV revealed that their respective CRPs are essential for disease symptom development and viral accumulation in citrus plants. In summary, our findings indicate that CiYMaV and CYVCV CRPs act as pathogenicity determinants, thereby enhancing our understanding of the functional repertoire within the mandariviruses proteome and providing a target for citrus hosts to defend against mandariviruses.

**IMPORTANCE:**

Mandariviruses, infecting a wide range of citrus varieties, cause serious epidemics in Pakistan, India, Turkey, China, Iran, Italy, and America. However, little information is available about pathogenicity mechanisms of mandariviruses. Here, we confirmed the importance of two mandarivirus CRPs of citrus yellow mottle-associated virus (CiYMaV) and citrus yellow vein clearing virus (CYVCV) in disease symptom development and viral accumulation in citrus plants. Our study first provides evidence that CiYMaV and CYVCV CRPs, nonstructural proteins, act as pathogenicity determinants with multiple functions. This offers a broad understanding of functional repertoire within the mandariviruses proteome. Further investigation of the underlying mechanisms of how CRP, as a virulence factor, modulates plant immunity may suggest a possible new strategy for combating mandarivirus infection in the field.

## INTRODUCTION

Citrus, a perennial woody plant with diverse varieties, is cultivated worldwide as a commercial fruit crop and is frequently challenged during cultivation by viral pathogens. The subgenus *Mandarivirus*, which belongs to the genus *Potexvirus* within the family *Alphaflexiviridae*, consists of three viral members: citrus yellow mottle-associated virus (*Potexvirus citriflavimaculae*)*,* citrus yellow vein clearing virus (*P. citriflavivenae*)*,* and Indian citrus ringspot virus (*P. citrindicum*). These viruses can be transmitted through grafting, and CYVCV can also be disseminated via the citrus whitefly and aphids (1–4). Mandariviruses have the ability to infect a wide range of citrus varieties, causing severe disease symptoms and posing a significant threat to the citrus industry in Asian, Middle Eastern regions, Italy, and America (5–7). However, the pathogenicity mechanisms of mandariviruses remain largely unknown due to the long growth cycle of their natural hosts and the lack of a rapid, efficient, and stable genetic transformation system in these hosts.

The mandarivirus genome is composed of a single-stranded, positive-sense RNA with a 3′ poly (A) tail and contains six open reading frames (ORFs) (5, 8). ORF1 potentially encodes a replication-associated polyprotein (REP); the ORF2/3/4 encode triple gene block proteins (TGB-1/2/3); ORF5 encodes the capsid protein (CP); ORF6, partially overlapping with ORF5, putatively encodes a cysteine-rich protein (CRP) which represents a crucial characteristic distinguishing mandarivirus from other viruses in the genus. Viral CRPs are known to be multifunctional proteins, serving as both an RNA silencing suppressor (RSS) and a pathogenicity determinant. For example, the CRP encoded by sweet potato chlorotic fleck virus (SPCFV NaBP) can enhance the pathogenicity of potato virus X (PVX) in *Nicotiana benthamiana*. It also functions as an RSS to suppress both local and systemic RNA silencing induced by either single-stranded or double-stranded RNA (ssRNA/dsRNA) (9). Chrysanthemum virus B (CVB) CRP (p12) is a weak RSS of post-transcriptional gene silencing (PTGS), and it also acts as a eukaryotic transcription factor to induce hyperplasia and severe leaf malformation in Chrysanthemum (*Chrysanthemum morifolium*) (10, 11). Phosphorylation of CRP encoded by Chinese wheat mosaic virus (CWMV) facilitates viral infection in plants by disrupting the function of RNA-binding protein UBP1-associated protein 2C (TaUBA2C) (12). Although these studies have demonstrated that several CRPs are related to viral pathogenicity in herbaceous hosts, the functions of viral CRPs encoded by woody plant viruses, such as mandarivirus CRPs, remain unclear.

In this study, we demonstrated that the presence of CiYMaV and CYVCV CRPs can exacerbate the PVX-induced symptoms and increase viral accumulation in *N. benthamiana*. We also revealed that the zinc finger (ZF) motif is crucial for CiYMaV CRP to effectively induce cell death in *N. benthamiana* leaves and that CYVCV CRP functions as an RSS to suppress local RNA silencing triggered by ssGFP but not dsGFP. Furthermore, the full-length cDNA infectious clones of CYVCV were constructed, resulting in the development of symptoms such as vein clearing, yellowing, leaf distortion, and dwarfing in Eureka lemon (*Citrus limon*). The mutational analysis demonstrated that CRP contributes to the infection of CiYMaV and CYVCV, and its characteristic motifs, nuclear localization signal (NLS) and ZF, are involved in the development of viral symptoms and viral accumulation. Collectively, these findings revealed that CiYMaV and CYVCV CRPs are vital pathogenic factors with multiple functions, promoting virus infection.

## RESULTS

### Sequence analysis of the CRP proteins encoded by mandariviruses

**Fig 1:**
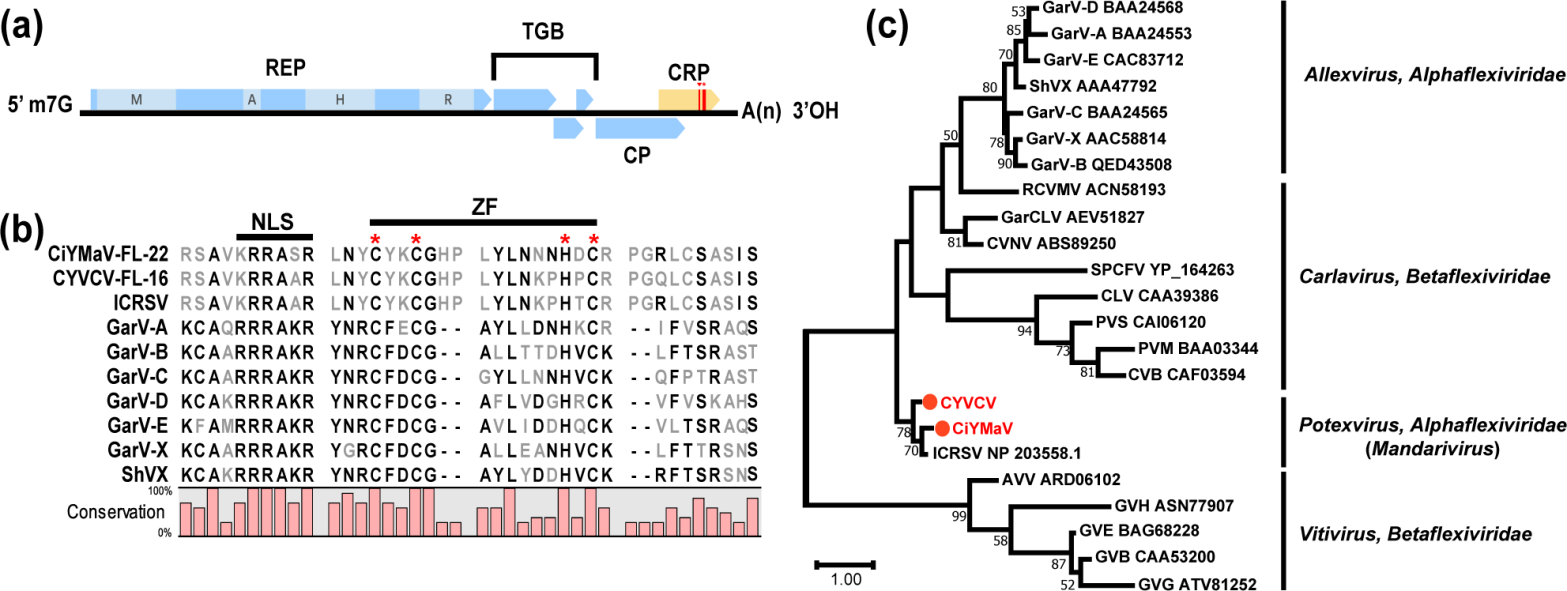
Sequence analysis of the mandarivirus CRPs. (**a**) A schematic diagram of mandarivirus genomic architecture with six open reading frames (ORFs). The CRP ORF is located on the 3-terminal portion of the mandarivirus genome, overlapping with the coat protein ORF. The nuclear localization signal (NLS) (red arrowhead) and zinc finger (ZF) (red asterisk) motifs are highlighted by red vertical bars. (**b**) Multiple alignment of the protein sequences of mandarivirus CRPs. NLS and ZF (red asterisk) motifs are overlined. Note that only partial sequences are displayed to highlight these regions. (**c**) Phylogenetic analysis of CRP amino acid sequences from 23 representative members of the families *Alphaflexiviridae* and *Betaflexiviridae*. The analysis was conducted with the MEGA 11 software using the maximum likelihood method with 1,000 bootstrap replications. The percentages for the bootstrap value less than 50% are not shown.

The mandarivirus CRP ORFs encode proteins consisting of 223 amino acids, which partially overlaps with the C-terminus of the CP ORF (Fig. 1a). To investigate the conservation of mandarivirus CRPs, sequence comparison of the mandarivirus CRPs was performed using NCBI BlastP. This analysis showed that the amino acid sequence identity of mandarivirus CRPs is in the range of 70%–80% and has the highest amino acid sequence identity with members of the genus *Allexivirus* though the identity was less than 40%. Amino acid sequence alignment showed that mandarivirus CRPs and allexivirus CRPs contain two conserved motifs, NLS and ZF, which exhibit characteristic features of eukaryotic transcription factors (Fig. 1b). To further investigate the evolutionary relationships of mandarivirus CRPs, a phylogenetic tree containing CRP amino acid sequences from 23 representative species of the families *Alphaflexiviridae* and *Betaflexiviridae* was constructed using the maximum likelihood method with MEGA 11. The result indicated that mandarivirus CRPs clustered into a single clade, with the closest genetic distance to allexivirus CRPs from the family *Alphaflexiviridae* and carlavirus CRPs from the family *Betaflexiviridae* (Fig. 1c).

### CiYMaV and CYVCV CRPs are pathogenicity determinants in *N. benthamiana*

**Fig 2:**
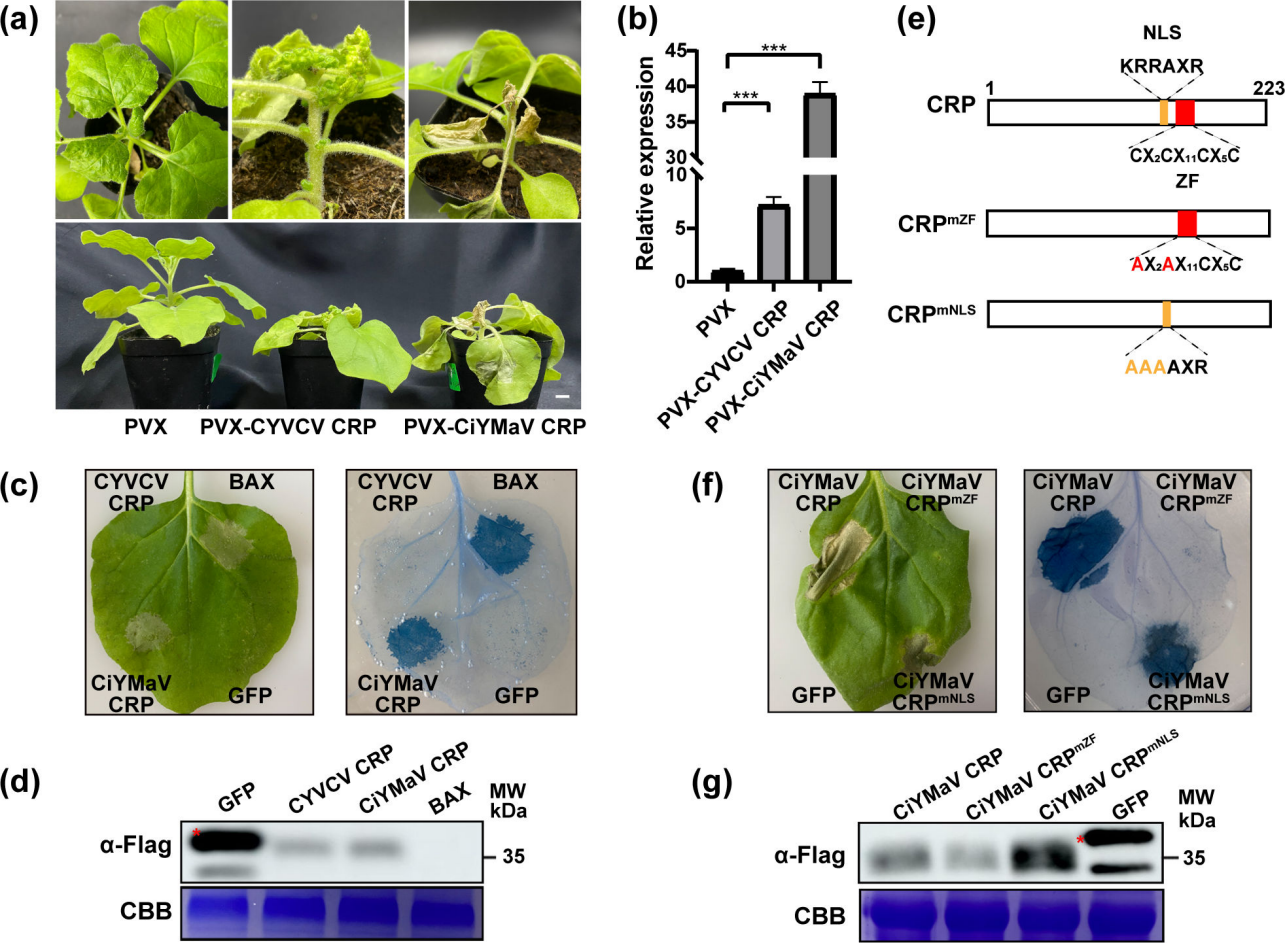
CiYMaV and CYVCV CRPs are major pathogenicity determinants in *Nicotiana benthamiana*. (**a**) Viral symptoms on *N. benthamiana* plants inoculated with PVX, PVX-CiYMaV CRP or PVX-CYVCV CRP at 15 days post-inoculation (dpi). Bar = 1 cm; (**b**) analysis of the relative expression levels of PVX genomic RNA in the systemic leaves of the PVX, PVX-CiYMaV CRP, or PVX-CYVCV CRP-infected plants through quantitative reverse transcription PCR (RT-qPCR). Error bars represent SD (*n* = 3). ***P < 0.001, Student’s *t*-test. This experiment was repeated three times with similar results. (**c**) Symptoms of *N. benthamiana* leaves inoculated with CiYMaV CRP-Flag, CYVCV CRP-Flag, BAX (positive control) and GFP-Flag (negative control). A typical leaf was photographed and stained with trypan blue at 2 dpi. (**d**) Western blot analysis of proteins in *N. benthamiana* transiently expressing CiYMaV CRP-Flag, CYVCV CRP-Flag or GFP-Flag. Coomassie Brilliant Blue (CBB) R250 staining was used as control. (**e**) Schematic diagrams of the alanine site-directed mutagenesis of CiYMaV CRP. The positions of the nuclear localization signal (NLS) motif were shown in yellow, and the ZF motif was shown in red. The amino acid residues of the NLS motif in red letters and the ZF motif in yellow letters were replaced with alanines. (**f**) Symptoms of *N. benthamiana* leaves inoculated with CiYMaV CRP-Flag, CiYMaV CRP^mNLS^-Flag, CiYMaV CRP^mZF^-Flag, and GFP-Flag. Cell death symptoms were further observed by trypan blue staining. Photographs were taken at 4 dpi. (g) CiYMaV CRP and its mutant proteins transiently expressed in *N. benthamiana* were analyzed by western blot. CBB R250 staining was used as control. The red asterisks (*) indicate the predicted protein sizes (**d and g**).

PVX-derived vector is widely used to systematically express viral genes of interest, and then virulence activity of these viral genes is determined by quantifying the impact on PVX pathogenicity. To explore the virulence of mandarivirus CRPs, CiYMaV and CYVCV CRPs were introduced into a PVX-derived vector for ectopic expression. *Agrobacterium tumefaciens* cultures carrying several PVX constructs (PVX-CiYMaV CRP, PVX-CYVCV CRP, or PVX-empty vector) were inoculated into *N. benthamiana* plants. At 10 dpi, the upper leaves of plants inoculated with PVX-CiYMaV CRP exhibited mosaic, malformation, and mild necrosis symptoms. Subsequently, these leaves displayed more severe necrosis symptoms at 15 dpi (Fig. 2a). Mosaic and mild malformation symptoms appeared on systemic leaves of PVX-CYVCV CRP-inoculated plants at 10 dpi, with further development of malformation at 15 dpi. Moreover, PVX-CiYMaV/CYVCV CRP-inoculated plants exhibited strong dwarfing symptoms at 15 dpi. In contrast, PVX-empty vector-inoculated plants displayed mild chlorosis and mosaic symptoms (Fig. 2a).

To further ascertain whether the severe symptoms observed in the presence of CRPs are caused by a higher accumulation of PVX, reverse transcription-quantitative PCR (RT-qPCR) was performed to analyze the level of PVX genome at 10 dpi. Compared with plants inoculated with the PVX-empty vector, PVX genome accumulation was significantly increased in those infiltrated with either PVX-CiYMaV CRP or PVX-CYVCV CRP (Fig. 2b). In contrast to PVX-CYVCV CRP, PVX-CiYMaV CRP caused a higher level of PVX genome accumulation and more severe symptoms, indicating a positive correlation between genome accumulation and PVX pathogenicity (Fig. 2a and b). Taken together, these results suggested that CiYMaV and CYVCV CRPs are pathogenicity determinants that enhance the pathogenicity of PVX.

To identify whether CiYMaV CRP could induce cell death in *N. benthamiana*, the coding sequence of CiYMaV or CYVCV CRPs was inserted into the plant binary expression vector pCAMBIA1300-Flag respectively, followed by agroinfiltration into *N. benthamiana*. BAX (a pro-apoptotic protein of the BCL-2 family) and GFP-Flag were used as the positive and negative controls, respectively. As shown in Fig. 2c, CiYMaV CRP triggered cell death in *N. benthamiana* leaves at 2 dpi similar to the positive control BAX. No necrosis symptoms were observed on leaves inoculated with CYVCV CRP or GFP-Flag. Western blot results revealed that the fusion proteins of CiYMaV CRP and CYVCV CRP were normally expressed (Fig. 2d). Next, we explored which domain of CiYMaV CRP is responsible for inducing necrosis. Based on the two conserved motifs of mandarivirus CRP, we generated two mutants of CiYMaV CRP, with alanines replacing either the first three amino acid residues of the NLS motif (CiYMaV CRP^mNLS^) or two adjacent cysteine residues of the ZF motif (CiYMaV CRP^mZF^) (Fig. 2e). The two CiYMaV CRP mutants were infiltrated into *N. benthamiana* leaves to detect its ability to induce cell death. As illustrated in Fig. 2f, g and n, *benthamiana* leaves expressing CiYMaV CRP and CiYMaV CRP^mNLS^ exhibited cell death symptoms at 2 dpi, whereas CiYMaV CRP^mZF^ did not induce cell death even at 4 dpi. These results indicated that the ZF motif is necessary for CiYMaV CRP to induce cell death in *N. benthamiana*.

### CYVCV CRP blocks RNA silencing induced by single-stranded RNA, but not double-stranded RNA in *N. benthamiana*

**Fig 3:**
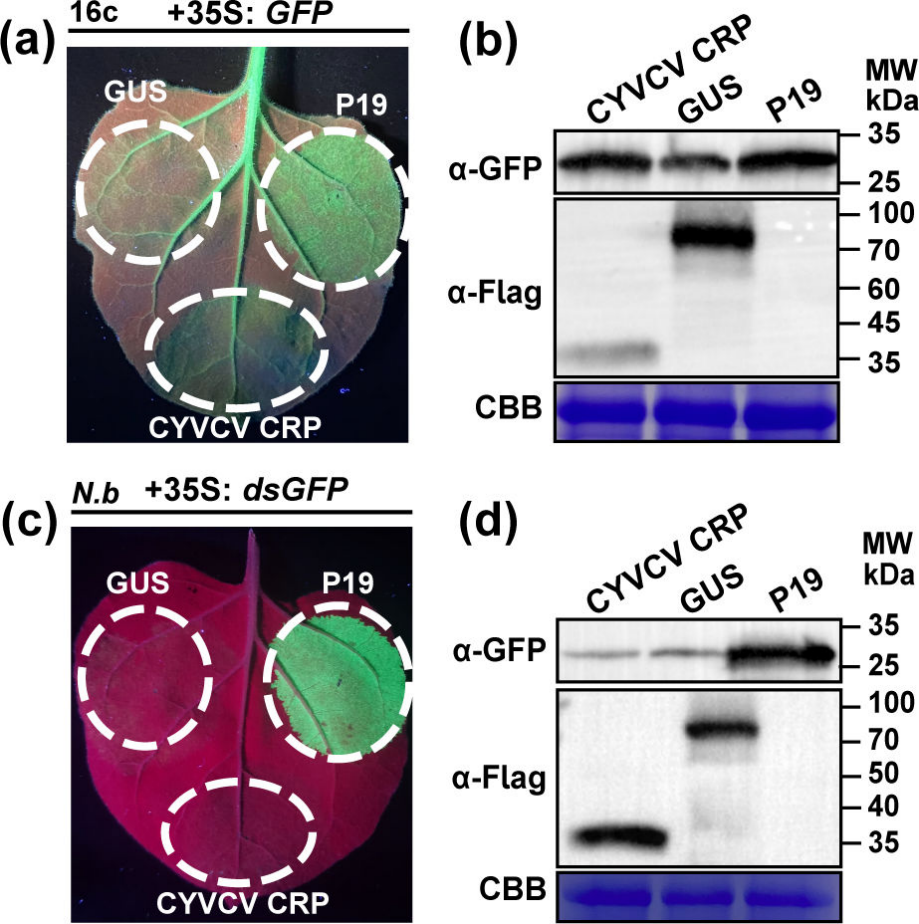
CYVCV CRP blocks local post-transcriptional gene silencing triggered by single-stranded GFP (sGFP) but not double-stranded GFP (dsGFP). (**a**) *GFP*-transgenic *Nicotiana benthamiana* (line 16c) leaf patches co-infiltrated with *Agrobacterium tumefaciens* cultures carrying 35S: *GFP* and CYVCV CRP, tomato bushy stunt virus (TBSV) P19 (positive control) or GUS (negative control). Photographs were taken under UV light at 3 days post-inoculation (dpi); (**b**) Western blot analysis of the accumulation level of GFP protein in agroinfiltrated leaf patches as indicated in (**a**); (**c**) *N. benthamiana* leaf patches co-infiltrated with *A. tumefaciens* cultures carrying 35S: *GFP* and 35S: ds*GFP* and CYVCV CRP, TBSV P19 or GUS. Infiltrated leaves were photographed under UV light at 4 dpi; (**d**) Western blot of GFP accumulation in agroinfiltrated leaf patches as indicated in (**c**). Coomassie Brilliant Blue (CBB) R250 staining was used as control (**b, d**).

As a multifunctional protein, CRP of many plant viruses can serve not only as a pathogenicity determinant but also as a viral suppressor of RNA silencing (VSR) (9, 11, 13, 14). Conducting continuous GFP fluorescence monitoring experiments on CiYMaV CRP is not feasible because it triggered cell death in *N. benthamiana*. Here, we verified the ability of CYVCV CRP to suppress RNA silencing using agroinfiltration. *A. tumefaciens* cultures expressing CYVCV CRP, TBSV P19 (positive control) or GUS (negative control) were combined with *A. tumefaciens* cultures carrying 35S: *GFP* in a ratio of 1:1 and subsequently infiltrated into *GFP*-transgenic *N. benthamiana* (line 16c) leaves. At 3 dpi, GFP fluorescence intensity substantially decreased in leaf patches co-infiltrated with 35S: *GFP* + GUS but was maintained in patches co-infiltrated with 35S: *GFP* + CYVCV CRP or 35S: *GFP* + P19, with the latter showing stronger fluorescence intensity (Fig. 3a). Western blot analyses verified that the accumulation of GFP proteins was higher in leaf patches co-infiltrated with 35S: *GFP* + CYVCV CRP or 35S: *GFP* + P19 than in those co-infiltrated with 35S: *GFP* + GUS (Fig. 3b). These results suggested that CYVCV CRP suppresses ssRNA-triggered local RNA silencing.

To test whether CYVCV CRP inhibited dsRNA-induced RNA silencing, *A. tumefaciens* cultures carrying 35S: *GFP* and 35S: ds*GFP* (a dsRNA targeting GFP) were mixed with CYVCV CRP, P19, or GUS in a ratio of 1:1:1 and infiltrated into *N. benthamiana* leaves. As shown in Fig. 3c, the infiltrated leaf patches with CYVCV CRP or GUS became red due to host RNA silencing, while GFP fluorescence was significantly enhanced in leaf patches co-infiltrated with P19. Visual assessment of GFP fluorescence was further corroborated by western blot at the molecular level (Fig. 3d). Taken together, these results indicated that CYVCV CRP effectively inhibits ssGFP but not dsGFP-induced local RNA silencing.

### CRP is essential for CiYMaV and CYVCV infection in citrus

**Fig 4:**
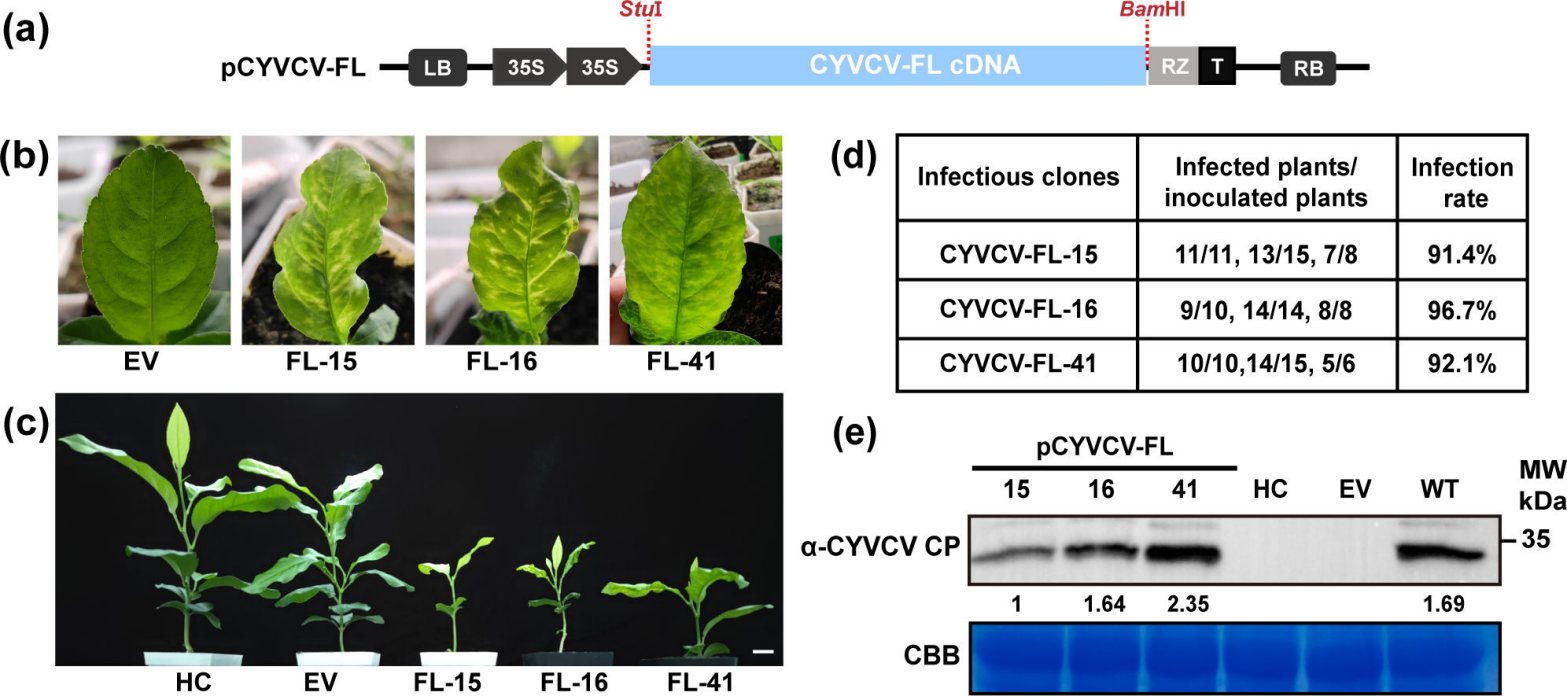
Construction and recovery of recombinant CYVCV full-length cDNA clones. (**a**) Schematic diagram showing the strategy for constructing the full-length cDNA clones of CYVCV. The CYVCV genome was cloned into the binary vector pCass4-Rz by *Stu*I and *Bam*HI sites. LB, left border sequence; 35S, cauliflower mosaic virus (CaMV) 35S promoter; RZ, ribozyme from satellite tobacco ringspot virus; T, a Nos terminator; RB, right border sequence; (**b, c**) symptoms of Eureka lemon (*Citrus limon*) seedlings inoculated with pCYVCVs (FL-15, 16, and 41) at 40 days post-inoculation (dpi). Bar = 5 cm; (**d**) analysis of the infection rates of the full-length cDNA clones of CYVCV using DTBIA and RT-PCR. The results were obtained through three independent experiments and expressed in terms of the mean value; (**e**) Western blot analysis of CYVCV coat protein accumulation with specific anti-1E1 antibody in systemically infected leaves as indicated in (**a**). Coomassie Brilliant Blue R250 staining was used as a loading control. HC, healthy control; EV, empty vector (pCass4-Rz)-infected control; WT, CYVCV-infected positive control.

We previously constructed the infectious clone of CiYMaV (15). Here, we constructed three full-length cDNA clones of CYVCV (pCYVCV-FL-15, 16, and 41) (Fig. 4a). These clones were separately agroinfiltrated into Eureka lemon (*C. limon*) seedlings using the vacuum infiltration method, and the empty vector (EV) was used as a negative control. At 40 dpi, the plants inoculated with pCYVCV-FL-15 and pCYVCV-FL-41 exhibited symptoms of leaf yellowing, mottling, and slight deformity. The plants inoculated with pCYVCV-FL-16 displayed severe vein clearing, yellowing, deformity, and dwarfing symptoms, resembling the typical symptoms induced by CYVCV (Fig. 4b and c). In contrast, no obvious symptoms were observed in healthy and EV-inoculated plants. Direct tissue blot immunoassay (DTBIA) and RT-PCR results confirmed that pCYVCV-FLs (15, 16, and 41) efficiently infected Eureka lemon plants with high infection rates (>91%), while healthy and EV-inoculated plants tested negative for CYVCV (Fig. 4d). Western bolt analysis of CYVCV CP showed that the accumulation of pCYVCV-FL-41 in upper leaves from the infected plants was higher than that detected in leaves infected with the other two pCYVCV-FLs (Fig. 4e). Based on these results, pCYVCV-FL-16 (hereinafter referred to as CYVCV) was selected for subsequent experiments.

**Fig 5:**
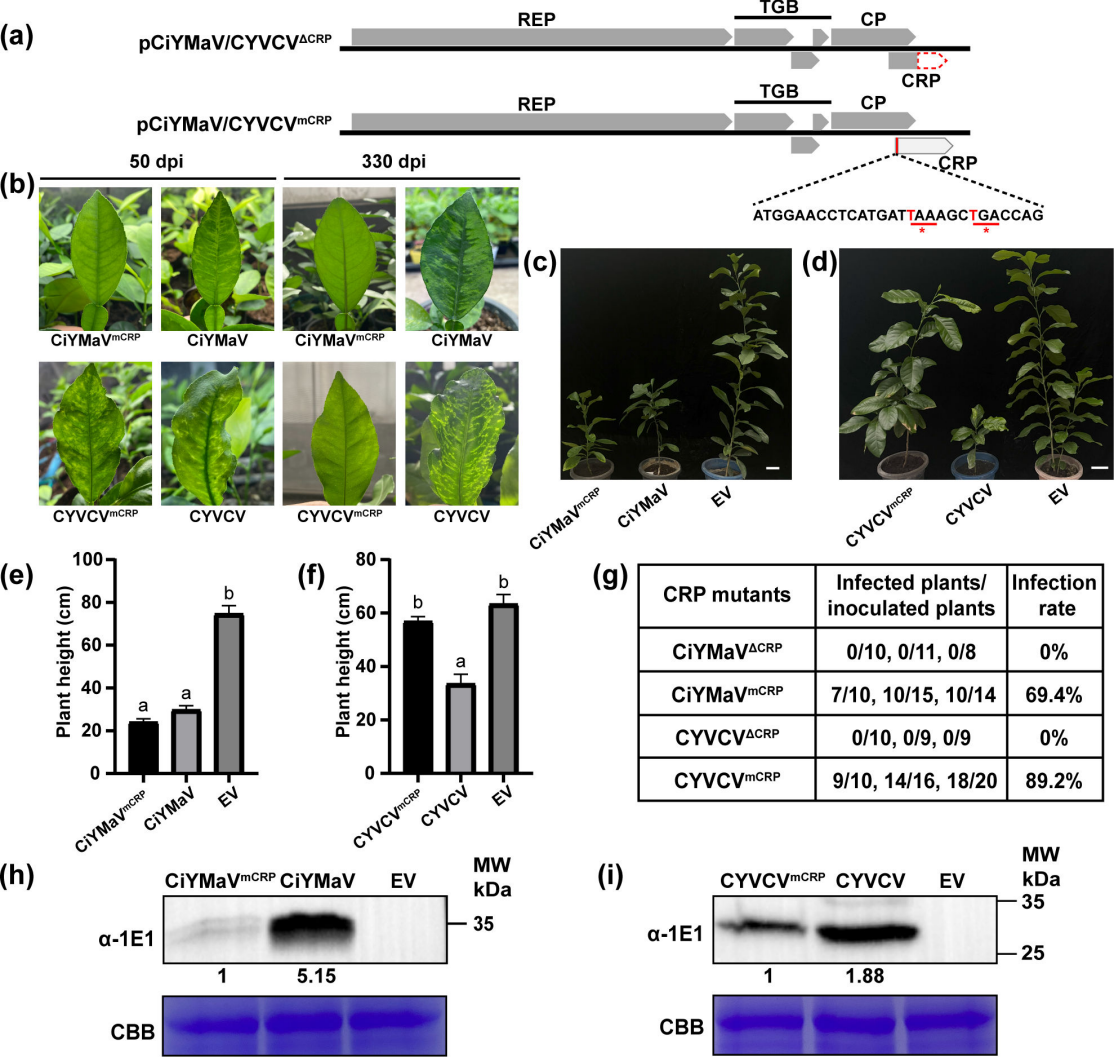
CRP plays an important role in the pathogenic process of CiYMaV and CYVCV in citrus. (**a**) Schematic diagrams of CRP mutants construction. Red dashed box indicates the deletion regions in the CRP deletion mutant (ΔCRP). The red vertical line indicates the position of the added stop codon (shown as two red asterisks) downstream of the CRP start codon (mCRP). (**b, c**) Symptoms of Chandler pummelo (*Citrus grandis*) inoculated with CiYMaV and CiYMaV^mCRP^ mutant. Photographs were taken at 50 days post-inoculation (b upper left) and 330 dpi (b upper right, **c**); (**b, d**) Phenotypes of Eureka lemon (*C. limon*) inoculated with CYVCV and CYVCV^mCRP^ mutant. Photographs were taken at 50 dpi (b lower left) and 330 dpi (b lower right, **d**). CYVCV, indicated the infectious clones of CYVCV FL-16. Bar = 5 cm; (**e, f**) Statistics of the plant height of Chandler pummelo inoculated with CiYMaV^mCRP^ and Eureka lemon inoculated with CYVCV^mCRP^ at 330 dpi. Different lowercase letters above the bars indicate statistically significant differences between the treatments group as determined by one-way ANOVA analysis followed by Tukey’s multiple comparisons test (*P* < 0.05). Error bars represent SEM; (**g**) analysis of the infection efficiency of CiYMaV^mCRP^, CiYMaV^ΔCRP^, CYVCV^mCRP^, and CYVCV^ΔCRP^ using DTBIA and RT-PCR. Data are shown as the mean value of three independent biological experiments; (**h, i**) Western blot analysis of CiYMaV and CYVCV coat protein accumulation with specific anti-1E1 antibody in upper noninfiltrated leaves as indicated in (**b**) and (**d**). Coomassie Brilliant Blue (CBB) R250 staining was used as control.

We constructed two CRP-mutated infectious clones for CiYMaV and CYVCV (referred to as CiYMaV/CYVCV^ΔCRP^ and CiYMaV/CYVCV^mCRP^), which make them unable to produce CRP. As shown in Fig. 5a, two stop codons were introduced into the nucleotides downstream of the CRP ORF start codon in CiYMaV/CYVCV^mCRP^ (replacements of nucleotides in the start codon of the CRP ORF inevitably impact the resulting CP) and C-terminal 101 amino acids were deleted in CiYMaV/CYVCV^ΔCRP^. Although the CRP gene partially overlaps with the C-terminus of the *CP* gene, the amino acid sequence of CP in those CRP mutants has not changed. The resulting CiYMaV^CRP^ mutants (CiYMaV^ΔCRP^ and CiYMaV^mCRP^) were inoculated on Chandler pummelo (*C. grandis*) seedlings, and the CYVCV^CRP^ mutants (CYVCV^ΔCRP^ and CYVCV^mCRP^) were inoculated on Eureka lemon seedlings by utilizing vacuum infiltration of *Agrobacterium*. DTBIA and RT-PCR results confirmed that CiYMaV/CYVCV^ΔCRP^ mutants failed to infect citrus plants, and the infection rates of CiYMaV^mCRP^ and CYVCV^mCRP^ were 69.4% and 89.2%, respectively (Fig. 5g). Plants inoculated with CYVCV^ΔCRP^ did not show any leaf symptoms at 50 dpi and 330 dpi, which is different from the symptoms caused by CYVCV (Fig. 5b and f). In contrast, plants inoculated with CYVCV^mCRP^ displayed slight leaf yellowing, vein clearing, and deformity symptoms at 50 dpi, whereas those plants exhibited milder leaf yellowing symptoms and had a significantly higher plant height compared to those inoculated with CYVCV by 330 dpi (Fig. 5b, d and f). The integrity of the CRP-mutated codons was analyzed, and all point mutations were maintained. Western blot analysis showed that the virus accumulation in citrus plants inoculated with CiYMaV^mCRP^ or CYVCV^mCRP^ mutants was substantially lower than in those infected with CiYMaV or CYVCV (Fig. 5h and i). These results suggested that CRP of CiYMaV and CYVCV was required for virus accumulation and symptom development in citrus.

### NLS and ZF motifs of CRP in CiYMaV and CYVCV are involved in virus pathogenicity

**Fig 6:**
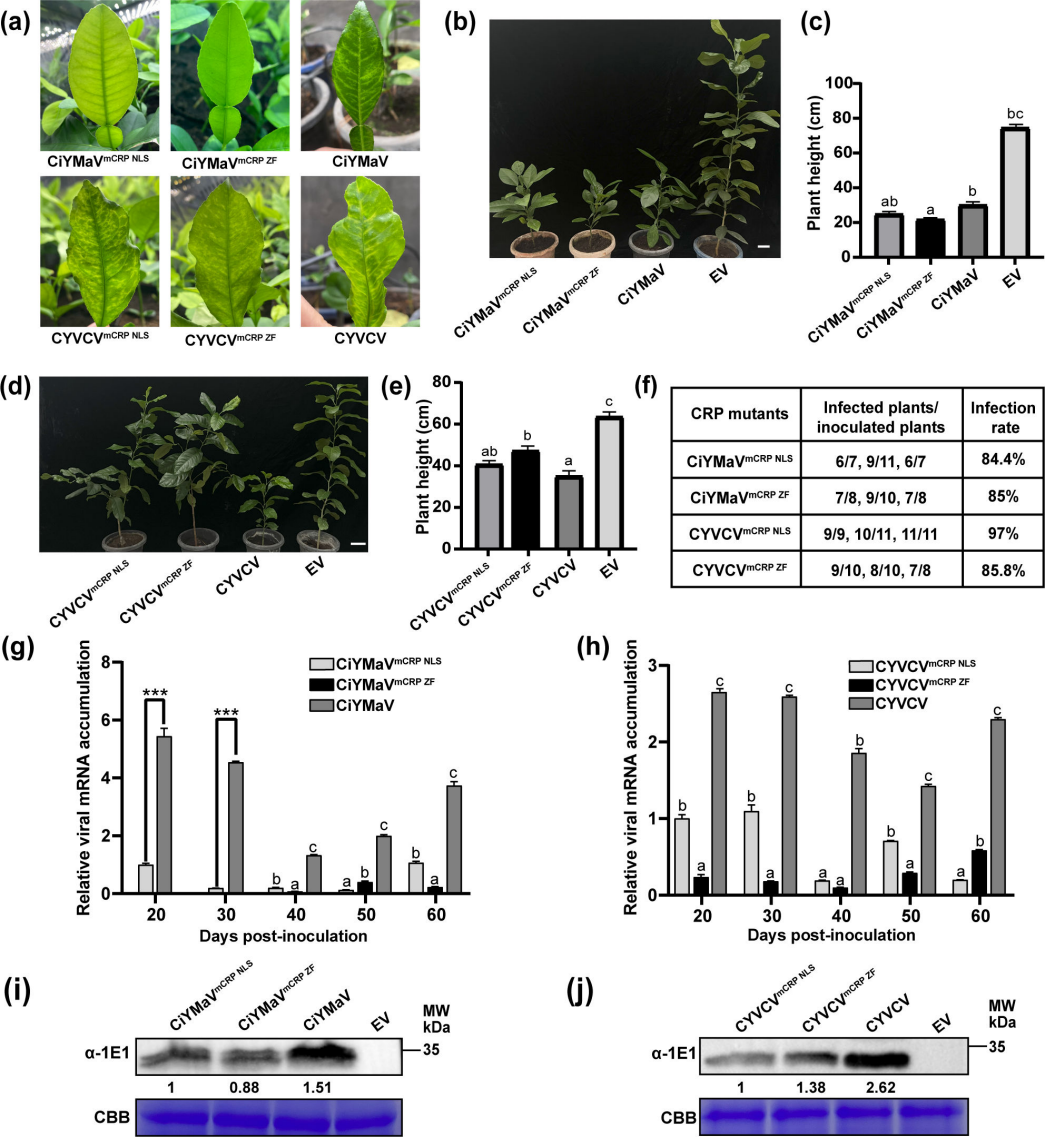
The NLS and ZF conserved motifs of CiYMaV and CYVCV CRP are involved in regulating virus accumulation and facilitating symptom formation. (**a, b**) Symptoms of Chandler pummelo (*Citrus grandis*) inoculated with CiYMaV, CiYMaV^mCRP NLS_,_^ and CiYMaV^mCRP ZF^ mutants. (**a, d**) Symptoms of Eureka lemon (*C. limon*) inoculated with CYVCV, CYVCV^mCRP NLS_,_^ and CYVCV^mCRP ZF^ mutants. Photographs were taken at 60 days post-inoculation (dpi, **a**) and 330 dpi (**b, d**). Bar = 5 cm; (**c, e**) Comparison of plant height of infiltrated citrus plants as indicated in (**b**) or (**d**). (**f**) Analysis of the infection efficiency of CiYMaV^mCRP NLS^, CiYMaV^mCRP ZF^, CYVCV^mCRP NLS^, and CYVCV^mCRP ZF^ at 60 dpi using DTBIA and RT-PCR. Data are shown as the mean value of three independent biological experiments. (**g, h**) Reverse transcription-quantitative PCR (RT-qPCR) data showed the relative expression level of viral genomic RNA in the systemic leaves of the CiYMaV, CYVCV, or its derived CRP mutant viruses-infected citrus plants over a time course. Student’s *t*-test was employed to statistically analyze the data of the CiYMaV group at 20 dpi and 30 dpi, ***P < 0.001. Different lowercase letters above the bars indicate statistically significant differences as determined by one-way ANOVA analysis followed by Tukey’s multiple comparisons test (*P* < 0.05). Error bars represent SEM (**g, h**). *csActin* was used as the internal reference. (**i, j**) Western blot analysis of CiYMaV and CYVCV coat protein accumulation in systemic leaves of citrus plants inoculated with CRP mutants of CiYMaV and CYVCV, using specific anti-1E1 antibody at 60 dpi. Coomassie Brilliant Blue (CBB) R250 staining was used as control.

To further investigate whether the NLS or ZF motifs of CRP are responsible for virus pathogenicity, CiYMaV/CYVCV^mCRP NLS^ and CiYMaV/CYVCV^mCRP ZF^ were constructed by alanine site-directed mutagenesis as we referred to Fig. 2e. CiYMaV^mCRP NLS^ and CiYMaV^mCRP ZF^ mutants were inoculated into Chandler pummelo seedlings, and CYVCV^mCRP NLS^ and CYVCV^mCRP ZF^ mutants were inoculated into Eureka lemon seedlings using vacuum infiltration of *Agrobacterium*. CiYMaV, CYVCV, and empty vector were employed as controls. At 60 dpi, the CiYMaV^mCRP NLS^ and CiYMaV^mCRP ZF^ mutants did not cause any leaf symptoms on Chandler pummelo, whereas plants inoculated with CiYMaV exhibited strong vein yellowing and mottling (Fig. 6a, upper panel). The CYVCV^mCRP NLS^ mutant induced mild mottling symptoms on the leaves of Eureka lemon, which gradually intensified over time (Fig. S1). By 60 dpi, the mutant inoculated plants displayed yellowing, vein clearing, and slight deformities, resembling those caused by CYVCV (Fig. 6a, lower panel). In contrast, the plants inoculated with the CYVCV^mCRP ZF^ mutant exhibited only mild mottling symptoms until 50 dpi (Fig. S1), indicating that the ZF motif of CYVCV CRP is crucial for symptom development. In addition, the plant height of CiYMaV^mCRP NLS^ and CiYMaV^mCRP ZF^ mutant-inoculated plants was significantly lower than that of the empty vector-inoculated plants but showed no significant difference compared to those inoculated with the CiYMaV at 330 dpi (Fig. 6b and c). However, compared to plants inoculated with CYVCV, the plant height of Eureka lemon plants inoculated with CYVCV^mCRP NLS^ and CYVCV^mCRP ZF^ mutants was significantly higher, but it remained lower than that of plants inoculated with empty vector (Fig. 6d and e). The results of DTBIA and RT-PCR showed that CiYMaV/CYVCV^mCRP NLS^ and CiYMaV/CYVCV^mCRP ZF^ mutants were capable of infecting citrus plants at 60 dpi, with an infection rate ranging from 85% to 97% (Fig. 6f). Meanwhile, the stability of the CRP mutated codons was assessed, and none of the mutation points were altered. The viral accumulation was determined by RT-qPCR from 20 to 60 dpi at 10-day intervals. The results showed that the levels of viral genomic RNA accumulation on citrus plants inoculated with CiYMaV^mCRP NLS^, CiYMaV^mCRP ZF^, CYVCV^mCRP NLS_,_^ or CYVCV^mCRP ZF^ mutants were significantly lower than those inoculated with CiYMaV or CYVCV (Fig. 6g and h). These results were further corroborated by western blotting, indicating that the NLS and ZF motifs of CRP in CiYMaV and CYVCV are important for virus accumulation in citrus plants (Fig. 6i and j). Together, our findings clearly demonstrated that the NLS and ZF motifs of CiYMaV and CYVCV CRP play crucial roles in determining virus pathogenicity.

## DISCUSSION

PTGS is an evolutionarily conserved defense mechanism that plays an essential role in the plant antiviral defense system (16). As a counter-defense strategy, most plant viruses have evolved to encode at least one RSS to counter the host’s antiviral defense (17). Many studies have indicated that CRPs of many plant viruses have RSS activity. For example, the CRP encoded by potato virus M (PVM), CWMV, CVB, and SPCFV can effectively inhibit local and/or systemic RNA silencing induced by ssRNA and/or dsRNA (9, 18–20). In this work, we found that CYVCV CRP possessed a weak activity inhibiting local silencing triggered by ssRNA but not dsRNA. Previous research has demonstrated that both the CP and CRP of allexivirus are RSS and interact with each other, but they possess the capacity to inhibit each other’s RSS activity (21). CYVCV CP has been reported to have strong RSS activity and interact with CYVCV CRP (22, 23). Therefore, it is possible that the roles of CYVCV CP and CRP in suppressing RNA silencing may be either antagonistic or synergistic.

Plant responses to phytopathogenic infection are often accompanied by a hypersensitive response (HR), shown by rapid and localized cell death (24). Many viral proteins cause cell death in *N. benthamiana*, such as papaya leaf curl virus (PaLCuV) V2, CVB P12, maize chlorotic mottle virus (MCMV) P31, and southern rice black-streaked dwarf virus (SRBSDV) P9-2 (14, 25–27). In this study, we found that CiYMaV CRP caused cell death in *N. benthamiana* when transiently expressed from a PVX-based vector or plant binary expression vector (Fig. 2a and c). However, CYVCV CRP did not induce necrotic symptoms, indicating that there are some fundamental differences in homologous proteins. Mutational analysis demonstrated that CiYMaV CRP without its ZF motif did not induce cell death, similar to CVB P12, indicating that the ZF motif is involved in HR induction. Additionally, considering that *N. benthamiana* does not serve as a host for CiYMaV, it is unlikely that this plant possesses an R protein specifically recognizing CiYMaV CRP, suggesting that the HR induction of CiYMaV CRP is caused by other mechanisms.

Numerous studies have revealed that viral CRPs also serve as a pathogenicity determinant, essential for the development of disease symptoms and the facilitation of virus infection. For example, ectopic expression of CVB P12 using tobacco mosaic virus (TMV) vector caused severe malformation and hyperplasia in *N. occidentalis* (10). The SPCFV NaBp heterologous expression induced chlorosis, necrotic mottling, and plant dwarfing, while also increasing the accumulation level of PVX genomic RNA in *N. benthamiana* (9). Here, we demonstrated that ectopic expression of CiYMaV and CYVCV CRPs using the PVX vector results in a higher level of PVX genome accumulation and more severe symptoms (Fig. 2a). However, the symptoms caused by these two mandarivirus CRPs exhibited divergence in *N. benthamiana*. Likewise, carlavirus CRPs were highly conserved, commonly sharing function as RSSs and enhancing the pathogenicity of PVX. But, the symptoms induced by carlavirus CRPs fall into two distinct types: leaf malformations and plant stunting. Hence, it is possible that the difference between these two CRPs arose from the sequence differences, given that their sequence identity is less than 80%, potentially leading to functional divergence. Alternatively, as previously reported, CYVCV CP functions as a viral pathogenicity factor and interacts with CRP (22, 23). CRP may facilitate the activity of CP, thereby contributing to viral pathogenicity.

Our previous research found that both CYVCV and CiYMaV caused plant dwarfing symptoms in citrus plants. Interestingly, compared with CYVCV, the CYVCV CRP mutants significantly alleviated the impact on plant dwarfing. However, the CiYMaV CRP mutants still exerted an influence on plant height similar to that of CiYMaV. Possible explanations for this apparent difference include the following. (i) Sequence divergence: the amino acid sequence identity between CYVCV and CiYMaV CRPs is less than 80%. Multiple site-specific differences between the two CRP proteins, particularly in the N-terminal region, may contribute to structural and functional divergence. (ii) Host specificity: the host ranges of CYVCV and CiYMaV differ. In this experiment, CYVCV was inoculated onto Eureka lemon seedlings, whereas CiYMaV was inoculated onto Chandler pummelo seedlings, which may have influenced the observed symptoms. (iii) Viral CRP function: the biological functions of viral CRPs may be influenced by the overall infection strategy of the virus and its specific interactions with the host. Further investigation into the mechanisms underlying CiYMaV-induced dwarfing would be valuable.

Many plant viruses belonging to filamentous (+) ssRNA viruses from diverse genera encode CRP, which is in the 3′-terminal ORF. CRP, which is characterized by NLS and ZF motifs, is unique to mandariviruses within potexviruses. It is possible that CRP has evolved to facilitate specific interactions between mandariviruses and their citrus hosts. In this work, our analysis found that CRPs encoded by viruses from diverse genera have low sequence homology. Interestingly, mandariviruses express an unusually long CRP, and two characterized motifs are located at the C-terminus of the protein, while those of other viruses are near the C-terminus. The enlarged CRP in mandarivirus could confer advantages in host interaction, functional diversification, or immune evasion, requiring further study. Overall, our study presents compelling evidence that mandarivirus CRPs are multifunctional proteins. Notably, this is the first report demonstrating that mandarivirus CRPs play important roles in disease symptom development and viral accumulation. With greatly reduced virulence, CiYMaV/CYVCV CRP mutants have great potential to be designed as viral vectors or provide cross-protection against aggressive isolates of the same virus. Additionally, the design of RNAi-based biopesticides or transgenic citrus targeting CRP could effectively mitigate viral pathogenicity. This comprehensive investigation provides a broad understanding of the function of mandarivirus CRPs, thereby offering valuable insights for establishing effective prevention and control strategies against mandarivirus. Accordingly, we need to continue exploring how CRP, as a virulence factor, modulates plant immunity and then interfere with CRP functions by designing targeted antiviral strategies to enhance resistance to mandarivirus infections.

## MATERIALS AND METHODS

### Plant materials and virus sources

Wild-type and GFP transgenic (line 16c) *N. benthamiana* were grown in a growth chamber at 25°C with 16 h (light): 8 h (dark) photoperiod. Eureka lemon and Chandler pummelo seedlings were maintained in illumination incubators set at 65% relative humidity, 16 h: 8 h light: dark, and 25°C: 20°C regimes. Etiolated citrus seedlings were grown in a growth chamber at 25°C with 0 h: 24 h light: dark photoperiod. The CiYMaV used in this study (CiYMaV-FL-22, GenBank accession number: PQ567900) has been described previously (15). CYVCV-infected citrus plants, previously collected in Yunnan Province, China (28), were maintained by grafting on Eureka lemon and placed in a greenhouse at the Citrus Research Institute, Chongqing, China.

### Sequence analysis

Mandarivirus CRP ORFs were predicted and identified using Open Reading Frame Finder software at the NCBI (https://www.ncbi.nlm.nih.gov/orffinder/). Multiple sequence alignments of mandarivirus CRPs amino acid sequences were performed using the CLC Genomics Workbench 11.0 software. Phylogenetic analysis based on the amino acid sequences of CRP proteins from 23 representative species was performed in MEGA 11 software by the maximum likelihood method using 1,000 bootstrap replications, with the percentages for the bootstrap value less than 50% not shown. The virus name and GenBank accession number in multiple alignment and phylogenetic tree are as follows: AVV, arracacha virus V (ARD06102); CiYMaV, citrus yellow mottle-associated virus (PQ567900); CLV, carnation latent virus (CAA39386); CVB, chrysanthemum virus B (CAF03594); CVNV, coleus vein necrosis virus (ABS89250); CYVCV, citrus yellow vein clearing virus (PQ567902); GarCLV, garlic common latent virus (AEV51827); GarV-A-E and X, garlic virus A-E and X (BAA24553, QED43508, BAA24565, BAA24568, CAC83712, and AAC58814); GVB, GVE, GVG, GVH, Grapevine virus B, E, G, H (CAA53200, BAG68228, ATV81252, ASN77907); ICRSV, Indian citrus ringspot virus (NP 203558); PVM, PVS, potato virus M, S (BAA03344, CAI06120); RCVMV, red clover vein mosaic virus (ACN58193); ShVX, shallot virus X (AAA47792); and SPCFV, sweet potato chlorotic fleck virus (YP_164263).

### Plasmid construction

For systematic expression of viral proteins in *N. benthamiana*, the coding sequences of CiYMaV and CYVCV CRPs were amplified from plasmid CiYMaV and the cDNA of CYVCV using Q5-Hot start high-fidelity DNA Polymerase (NEB, Beijing, China). Then, the resulting products were digested with *Cla*I and *Sal*I and were inserted into the pGR106 vector (a PVX-derived vector) to generate constructs PVX-CiYMaV CRP and PVX-CYVCV CRP. To generate the constructs to transiently express CiYMaV CRP-Flag and CYVCV CRP-Flag, the full-length CiYMaV and CYVCV CRPs were cloned and recombined into pCV1300-Flag (a plant binary expression vector). The pCV-CiYMaV CRP-Flag plasmid was employed as the template to generate two mutants (CiYMaV CRP^mNLS^-Flag and CiYMaV CRP^mZF^-Flag), wherein the NLS and ZF motifs were mutated, respectively.

To construct the full-length cDNA clones, the whole genome of CYVCV was amplified from the cDNA of CYVCV. Then, the PCR products were cloned into pCass4-Rz using the In-Fusion cloning system (Vazyme, Nanjing, China) to generate constructs pCYVCV-FLs (15, 16, and 41). To construct CiYMaV and CYVCV mutants that are incapable of producing CRP while not affecting the normal coding of other proteins, the partial sequences of CiYMaV and CYVCV were amplified to mutate the nucleotides following the start codons in the CRP gene to two TAA codons. The deletion mutants, expressing the N-terminal 101 amino acids of CRP, were subsequently amplified. The PCR fragments of CiYMaV were digested with *Eco*81I and *Bam*HI, and the PCR fragments of CYVCV were digested with *Sal*I and *Bam*HI and then inserted into the corresponding plasmid to generate mutants CiYMaV^mCRP^, CiYMaV^ΔCRP^, CYVCV^mCRP^, and CYVCV^ΔCRP^. Mutants CiYMaV^mCRP NLS^, CiYMaV^mCRP ZF^, CYVCV^mCRP NLS^, and CYVCV^mCRP ZF^ were generated by alanine substitution using a two-step overlap PCR. The primers used in this study are listed in Table S1 of the Supporting Information.

### Agroinfiltration assays

The recombinant PVX vectors expressing CiYMaV or CYVCV CRPs were transformed into *A. tumefaciens* strain GV3101 (pSoup). The recombinant pCV1300-Flag vector expressing CYVCV CRP-Flag, CiYMaV CRP-Flag, or its mutants was transformed into *A. tumefaciens* strain GV3101. The CiYMaV, CYVCV, and their mutants were transformed into *A. tumefaciens* strain EHA105. Pre-inoculum cultures of *A. tumefaciens* were incubated at 28°C for 48 h on Luria-Bertani (LB) solid medium (with 50 µg/mL kanamycin, 20 µg/mL rifampicin). Then, a single colony was selected and incubated overnight in LB broth (with 50 µg/mL kanamycin, 20 µg/mL rifampicin) at 28°C with shaking at 200 rpm. The cultures were centrifuged (6,000 *g*, 10 min) and subsequently resuspended in an infiltration buffer (containing 10 mM MES, pH 5.6, 10 mM MgCl_2_, 100 µM acetosyringone) to adjust the optical density (OD600) to 0.5–1.0. The cell suspensions should be incubated in the dark at 28°C for 3 h before infiltration. For transient expression, *A. tumefaciens* cultures were injected into leaves of 4–6 leaf stage *N. benthamiana* plants using a needle-less syringe.

The PTGS assay was conducted by agroinfiltration of 35S: *GFP* with GFP-Flag as a negative control, P19 as a positive control, or CYVCV-CRP-Flag into leaves of *GFP*-transgenic *N. benthamiana* (line 16c) at 4–6 leaf stage. For inverted repeat RNA-induced PTGS (IR-PTGS) assay, *A. tumefaciens* cultures harboring 35S: *GFP*, 35S: ds*GFP*, and either CYVCV CRP-Flag, P19 or GUS were combined in a 1:1:1 ratio and subsequently infiltrated into *N. benthamiana* leaves. For viral inoculation, *A. tumefaciens* cultures carrying the infectious clones of CiYMaV, CYVCV, or their mutants were infiltrated into 7-day-old etiolated citrus seedlings using the vacuum infiltration method, as previously described (15). Briefly, the citrus seedlings were immersed in cell suspensions, subjected to a vacuum of −1 standard atmosphere for 1 min.

### RNA extraction and RT-qPCR

Total RNA was extracted from the collected leaf tissues following TRIzol reagent (Invitrogen, San Diego, CA, USA). Then, 1 µg of total RNA was reverse transcribed to complementary DNA (cDNA) using All-In-One 5 × RT MasterMix (ABM, Shanghai, China). RT-qPCR was performed using BlasTaq 2 × qPCR Master Mix (ABM) on a qTOWER^3^ real-time system (Analytik Jena, Germany). Primers used for RT-qPCR are listed in Table S1. *N. benthamiana* elongation factor 1-alpha (*EF1-α*) gene (CN744397) and *C. sinensis* actin (*CsActin*) gene (GU911361.1) served as internal control. The relative gene expression levels were analyzed using the 2^−ΔΔCt^ method (29). The experiments contained three independent biological replicates (citrus plants under the same treatment were mixed and randomly divided into three groups as biological replicates), and four technical repeats were performed.

### Trypan blue staining

Trypan blue staining was performed as previously described by Shan et al. (30). Briefly, the infiltrated *N. benthamiana* leaves were harvested, fully immersed in a trypan blue staining solution (0.025% trypan blue in the mixture of phenol:lactic acid:ethanol [1:1:2 (vol/vol/vol)]) and then placed in boiling water for a duration of 10–15 min. After incubation at room temperature for 1 h, the leaf samples were destained overnight with 2.5 g/mL chloral hydrate on a horizontal shaker and then photographed.

### Detection using DTBIA and RT-PCR

The infectivity of the infectious clones of CYVCV and the mutants of CiYMaV and CYVCV was assessed using DTBIA and RT-PCR. DTBIA was conducted as previously described (5, 15). Concisely, tender shoots or petioles from infected plants were cut transversely, and then, the freshly cut surface was then gently pressed on nitrocellulose filter membranes (Bio-Rad, CA, USA). The monoclonal antibody 1E1 which exhibited positive reactivity with CiYMaV and/or CYVCV-infected samples, was utilized at a 1:3,000 dilution and incubated with membranes for 1.5 h (5, 15). Subsequently, the membranes were transferred to a 5% BSA solution containing 1:2,000 diluted alkaline phosphatase (AP)-conjugated goat anti-mouse IgG (IgG/AP). Finally, the membranes were incubated in nitrotetrazolium blue chloride/5-bromo-4-chloro-3-indolyl phosphate (NBT/BCIP) buffer (Promega, Madison, WI, USA) to visualize the detection. RT-PCR analysis was performed using the PrimeScript One-Step RT-PCR Kit Ver.2 (Takara) with specific primers CiYMaV-CP-F/R or CYVCV-CP-F/R (Table S1).

### Protein extraction and western blot analysis

Total protein was extracted from systemic leaves of inoculated plants with lysis buffer (containing 100 mM Tris-HCl pH 6.8, 10% sodium dodecyl sulfate (SDS), 2% β-mercaptoethanol) and separated by electrophoresis on 12.5% SDS polyacrylamide gels electrophoresis. Subsequently, the gels were stained by Coomassie Brilliant Blue R250 staining solution or transferred to polyvinylidene fluoride (PVDF) membranes (Bio-Rad) by the wet electroblotting method. The proteins were detected through immunoblots using Flag-specific antibody (1:5,000; TransGen, Beijing, China), GFP-specific antibody (1:5,000; TransGen), or CiTMaV/CYVCV-specific antibody 1E1 (1:3,000), followed by goat anti-mouse IgG (H + L) HRP-linked secondary antibody (1:10,000; TransGen). Finally, the hybridization signals were detected using the Omni-ECL pico light chemiluminescence kit (Epizyme, Shanghai, China), and imaging was performed using a Gel Doc XR + Gel Documentation system (Bio-Rad).

## Data Availability

The full-length sequences of the three CYVCV clones have been deposited in GenBank under accession numbers PQ567901, PQ567902, and PQ567903. The data used and/or analyzed to support the findings of this study are available from the corresponding author upon reasonable request.

## SUPPLEMENTAL MATERIAL

Figure S1 jvi.02237-24-s0001.tif The NLS and ZF conserved motifs of CYVCV and CiYMaV CRPs influence symptom development.

Table S1 jvi.02237-24-s0002.pdf Sequences of primers used in this study.

ASM does not own the copyrights to Supplemental Material that may be linked to, or accessed through, an article. The authors have granted ASM a non-exclusive, world-wide license to publish the Supplemental Material files. Please contact the corresponding author directly for reuse.
