# Supplementary figures and images for: The role of cysteine-rich protein in enhancing mandarivirus infectivity and pathogenicity
Source: J Virol. 2025 May 19;99(6):e02237-24. doi: 10.1128/jvi.02237-24 (PMC12172455; doi:10.1128/jvi.02237-24)

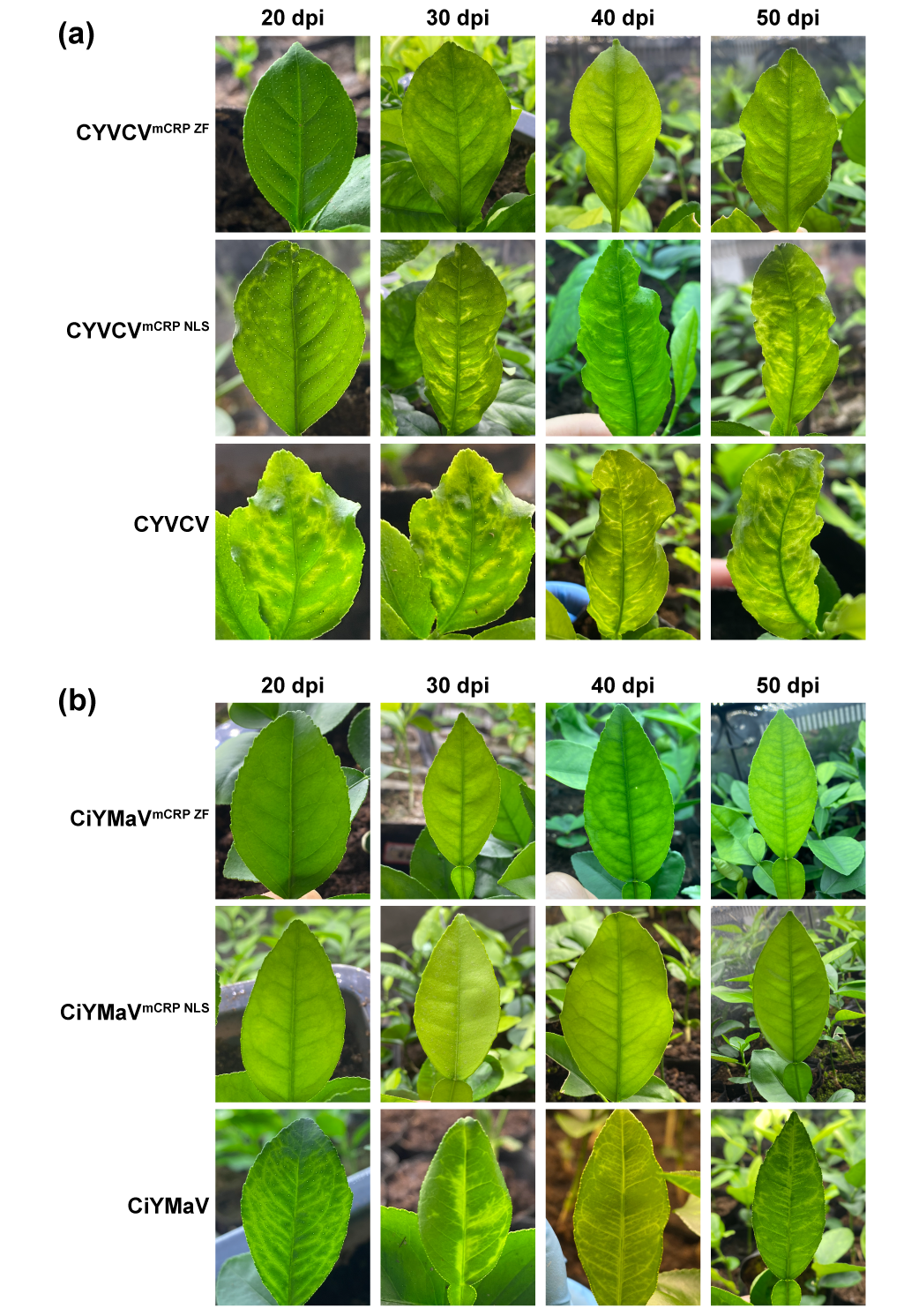

Supplement: Figure S1 — The NLS and ZF conserved motifs of CYVCV and CiYMaV CRPs influence symptom development. [file jvi.02237-24-s0001.tif]
