# Supplementary material for: The role of cysteine-rich protein in enhancing mandarivirus infectivity and pathogenicity
Source: J Virol. 2025 May 19;99(6):e02237-24. doi: 10.1128/jvi.02237-24 (PMC12172455; doi:10.1128/jvi.02237-24)
Supplement: Table S1 — Sequences of primers used in this study. [file jvi.02237-24-s0002.pdf]

SUPPLEMENTAL MATERIAL

FIGURE S1 The NLS and ZF conserved motifs of CYVCV and CiYMaV CRPs influence symptom development. (a) Symptoms on Chandler pummelo (*Citrus grandis*) inoculated with CiYMaV, CiYMaV$^{mCRP\ ZF}$ and CiYMaV$^{mCRP\ NLS}$ mutants. (b) Symptoms of Eureka lemon (*C. limon*) inoculated with CYVCV, CYVCV$^{mCRP\ NLS}$ and CYVCV$^{mCRP\ ZF}$ mutants. Photographs were taken at 20 days post-inoculation (dpi), 30 dpi, 40 dpi and 50 dpi.

TABLE S1 Sequence of primers used in this study

Primer name	Sequence (5’-3’)	Usage
PVX-CiYMaV CRP-F	CCATCGATATGGAACCTCATGATCAAAGC	To construct transient expression vectors for expressing CYVCV CRP and its CRP mutants
PVX-CiYMaV CRP-R	GCGTCGACTCATCTGGGGTCGAGGAGTTC	
pCA-CiYMaV CRP-F	CGACGACAAGACCGTCACCATGGAACCTCATGATC AAAGC	
pCA-CiYMaV CRP-R	GAGGAGAAGAGCCGTCGTCTGGGGTCGAGGAGTT CATG	
pCA-CiYMaV CRP-mNLS-overlap-1R	GGCTGCTGCTGCGACAGCAGAGCGACTTGAACC	
pCA-CiYMaV CRP-mNLS-overlap-2F	GTCGCAGCAGCAGCCTCTCGACTTAACTACTGT	
pCA-CiYMaV CRP-mZF-overlap-1R	TTTAGATAAAGTGGGTGCCCTGCCTTGTATGCGTA GTTAAGTCGAGAGGCTCG	
pCA-CiYMaV CRP-mZF-overlap-2F	ACCCACTTTATCTAAATAACAACGCAGACGCACGAC CAGGTCGTCTTTGTTCT	
PVX-CYVCV CRP-F	CCATCGATATGGAACCTCATGATCAAGGC	To construct transient expression vectors for expressing CYVCV CRP
PVX-CYVCV CRP-R	GCGTCGACTCATCTGGGGTCAAGGAGCTC	
pCA-CYVCV CRP-F	CGACGACAAGACCGTCACCATGGAACCTCATGATC AAGGC	
pCA-CYVCV CRP-R	GAGGAGAAGAGCCGTCGTCTGGGGTCAAGGAGCT CGTG	
CYVCV-FL-F	TCATTTCATTTGGAGAGGCCTGAAAAGCAAACATTA TCATACACACCC	To construct the full-length cDNA clones of CYVCV and its CRP mutants
CYVCV-FL-R	CGGTGACAGGGTATCGGATCCTTTTTTTTTTTTTTT TTTTTTTTTTCAGAAAA	
CYVCV-FL-16-SalI-1F	GCGTCGACGAGTACCCTCTAAGCCAGATACA	
CYVCV-FL-16-mCRP-overlap-1R	GGTCAGCCTTAATCATGAGGTTCCATACCACCC	
CYVCV-FL-16-mCRP-overlap-2F	TGATTAAGGCTGACCAGCCTCCAGCCAACTGGA	
CYVCV-FL-16-ΔCRP-1R	TTAGATGTTGAAAGGGGTCGGGC	

CYVCV-FL-16-ΔCRP-2R	CGCGGATCCTTTTTTTTTTTTTTTTTTTTTTTTTCAGAAAATGGAAACTGAAAGCCTGAATATTTATGTTTTAGATGTTGAAAGGGGTC	
CYVCV-FL-16-mCRP NLS-overlap-1R	CGCTGCTGCTGCGACAGCTGAGCGGCTGGT	
CYVCV-FL-16-mCRP NLS-overlap-2F	GTCGCAGCAGCAGCGGCTCGCCTGAACTAC	
CYVCV-FL-16-mCRP ZF-overlap-1R	CCCTGCCTTGTATGCGTAGTTCAGGCGAGCCGCTCTAC	
CYVCV-FL-16-mCRP ZF-overlap-2F	TACGCATACAAGGCAGGGCACCCCTTATATCTAAATAA	
CiYMaV-FL-22-eco81I-1F	ACCTGCCTGAGGGGTTTAAATCTAACTGAGTCCGA	To construction of CiYMaV CRP mutants
CiYMaV-FL-22-mCRP-overlap-1R	GGTCAGCTTTAATCATGAGGTTCCATACGACGCGG	
CiYMaV-FL-22-mCRP-overlap-2F	TGATTAAAGCTGACCAACCACCCGCGAACTGGATG	
CiYMaV-FL-22-ΔCRP-1R	CTAGGTGGCAAAAGGGGTCTTGC	
CiYMaV-FL-22-ΔCRP-2R	CGCGGATCCTTTTTTTTTTTTTTTTTTTTTTTTTTTTTTCAGAAAATGGAAACTGAAAGCCTGAATATTTATGTTCTAGGTGGCAAAAGGGGTCTT	
CiYMaV-FL-22-mCRP NLS-overlap-1R	GGCTGCTGCTGCGACAGCAGAGCGACTTGAACCCA	
CiYMaV-FL-22-mCRP NLS-overlap-2F	GTCGCAGCAGCAGCCTCTCGACTTAACTACTGTTA	
CiYMaV-FL-22-mCRP ZF-overlap-1R	CCCTGCCTTGTATGCGTAGTTAAGTCGAGAGGCTCGGC	
CiYMaV-FL-22-mCRP ZF-overlap-2F	TACGCATACAAGGCAGGGCACCCACTTTATCTAAATAA	
CiYMaV-FL-22-R	CGCGGATCCTTTTTTTTTTTTTTTTTTTTTTTTTTTTTTCAGAAAATGGAAACTGAAAGCCTG	
PVX-CP-qF	CGCAACAAATGAGGACCTCAGCAAT	For qRT-PCR
PVX-CP-qR	GCAGCCTGTGCCATAGTGTCTGTG	
CiYMaV-qF	GTATTGCGACCACCATCCCG	
CiYMaV-qR	TGGGTGCCGATTTCCCTTTA	
CYVCV-qF	TCCACCTGCCTTTAACCCG	
CYVCV-qR	GTTCAAGATGCGGACCCAAG	
NbEF1-α-qF	TGGTGTCCTCAAGCCTGGTATGGTTG	
NbEF1-α-qR	ACGCTTGAGATCCTTAACCGCAACATTCTT	
csActin-qF	CATCCCTCAGCACCTTCC	
csActin-qR	CCAACCTTAGCACTTCTCC	
CiYMaV-CP-F	ATGAGCTTAGACTATCAGCATCCC	For RT-PCR

CiYMaV-CP-R	CTAGGTGGCAAAAGGGGTCTTGC	detection
CYVCV-CP-F	ATGAGCTTCGACTACACTCACCCT	
CYVCV-CP-R	TTAGATGTTGAAAGGGGTCGGGCG	
